# Quantitative fluorescence angiography aids novice and experienced surgeons in performing intestinal resection in well-perfused tissue

**DOI:** 10.1007/s00464-021-08518-7

**Published:** 2021-05-03

**Authors:** Nikolaj Nerup, Morten Bo Søndergaard Svendsen, Jonas Hedelund Rønn, Lars Konge, Lars Bo Svendsen, Michael Patrick Achiam

**Affiliations:** 1 Department of Surgical Gastroenterology, Copenhagen University Hospital Rigshospitalet, Blegdamsvej 9 DK-2100, Copenhagen Ø, Denmark; 2 Copenhagen Academy for Medical Education and Simulation, Center for Human Resources and Education, Copenhagen, Capital Region of Denmark Denmark

**Keywords:** Image guided surgery, Near-infrared imaging, Fluorescence guided surgery, Anastomotic leakage, Indocyanine green angiography

## Abstract

**Background:**

Anastomotic leakage (AL) after gastrointestinal resection is a devastating complication with huge consequences for the patient. As AL is associated with poor blood supply, tools for objective assessment of perfusion are in high demand. Indocyanine green angiography (ICG-FA) and quantitative analysis of ICG-FA (q-ICG) seem promising. This study aimed to investigate whether ICG-FA and q-ICG could improve perfusion assessment performed by surgeons of different experience levels.

**Methods:**

Thirteen small bowel segments with a varying degree of devascularization, including two healthy sham segments, were constructed in a porcine model. We recruited students, residents, and surgeons to perform perfusion assessment of the segments in white light (WL), with ICG-FA, and after q-ICG, all blinded to the degree of devascularization.

**Results:**

Forty-five participants fulfilled the study (18 novices, 12 intermediates, and 15 experienced). ICG and q-ICG helped the novices correctly detect the healthy bowel segments to experienced surgeons’ level. ICG and q-ICG also helped novice surgeons to perform safer resections in healthy tissue compared with normal WL. The relative risk (RR) of leaving ischemic tissue in WL and ICG compared with q-ICG, even for experienced surgeons was substantial, intermediates (RR = 8.9, CI95% [4.0;20] and RR = 6.2, CI95% [2.7;14.1]), and experienced (RR = 4.7, CI95% [2.6;8.7] and RR = 4.0, CI95% [2.1;7.5]).

**Conclusion:**

Q-ICG seems to guide surgeons, regardless of experience level, to safely perform resection in healthy tissue, compared with standard WL. Future research should focus on this novel tool’s clinical impact.

The incidence of gastrointestinal cancer is increasing, and surgical resection remains an important part of the treatment of most cancers. The long-term survival highly depends on the postoperative outcome, and complications as anastomotic leakage (AL) greatly influence survival and recurrence rates in esophageal and colorectal resections [1–3]. The anastomotic healing process is complex and sufficient perfusion of the anastomotic tissue is important in each phase of the healing process [4]. Thus, low anastomotic perfusion has been associated with an increased risk of AL [5, 6].

Anastomotic perfusion is traditionally assessed visually by; the amount of bleeding from resection lines, the color of the tissue, presence of peristalsis, and manually by palpation of the mesenteric pulse. However, these assessment methods are limited in minimally invasive surgery, and reproducibility, sensitivity, and specificity rates are low [7]. A newer method using indocyanine green fluorescence angiography (ICG-FA) enables visualization of the microvasculature normally hidden from the human eye and has shown promising results [8].

Recently two randomized controlled studies investigating perioperative ICG-FA were published, but one failed to prove a significantly lower rate of AL with ICG-FA assisted colorectal resection (*n* = 240, *p* = 0.2) [9]. The other study determined a significantly reduced AL rate when using ICG-FA, but only in low rectal anastomoses [10]. Systematic reviews have shown that the use of ICG-FA for perfusion assessment seems to lower the AL rate in both esophageal [11] and colorectal resection [12]. However, both systematic reviews state that the included studies were heterogeneous in design and limited by bias. The conflicting results indicate a need for an objective and unbiased evaluation of ICG-FA.

Recently, quantitative analysis of ICG-FA (q-ICG) has been introduced to limit the observer bias and reduce reproducibility problems when normal non-quantitative visual ICG-FA is performed. We have previously described our quantification software’s validation, reliability, and reproducibility (q-ICG) in a series of animal studies in both normal, increased, and reduced perfusion [13–17].

In almost every study addressing perfusion assessment with or without the use of ICG, the surgeons assessing the perfusion have been very experienced, and the usability of ICG has not been questioned [11, 12]. Furthermore, very few studies have investigated the impact of surgical experience on performing accurate perfusion assessment during gastrointestinal resection.

The present study aimed to investigate if the use of ICG-FA and q-ICG would improve surgeons’ (with different experience level) accuracy in perfusion assessment compared with using conventional white light (WL).

## Methods

### Animal model

We used a porcine model (female, Danish Landrace, 33–36 kg), the experimental protocol was approved by The Danish Animal Experimentation Inspectorate (#2016-15-0201-01015), and the study performed following the European Union legislation on animal experimentation, and the ARRIVE Guidelines [18]. The animals were part of another study [15] in accordance with the principle of replacement, refinement, and reduction [19]. Animals were acclimatized in the experimental facility for two weeks. Premedication was either with tiletamine/zolazepam, and full anesthesia was induced with propofol and haldid. After tracheal intubation, gastric tubes, urinary catheters, venous and arterial catheters were placed. Close monitoring of mean arterial pressure, heart rate, oxygen saturation, expiratory breath carbon dioxide, arterial blood oxygenation, and carbon dioxide was achieved. Analgesia was provided throughout the experiment, and animals were euthanized at the end of the experiment by an overdose of pentobarbital.

**Fig. 1:**
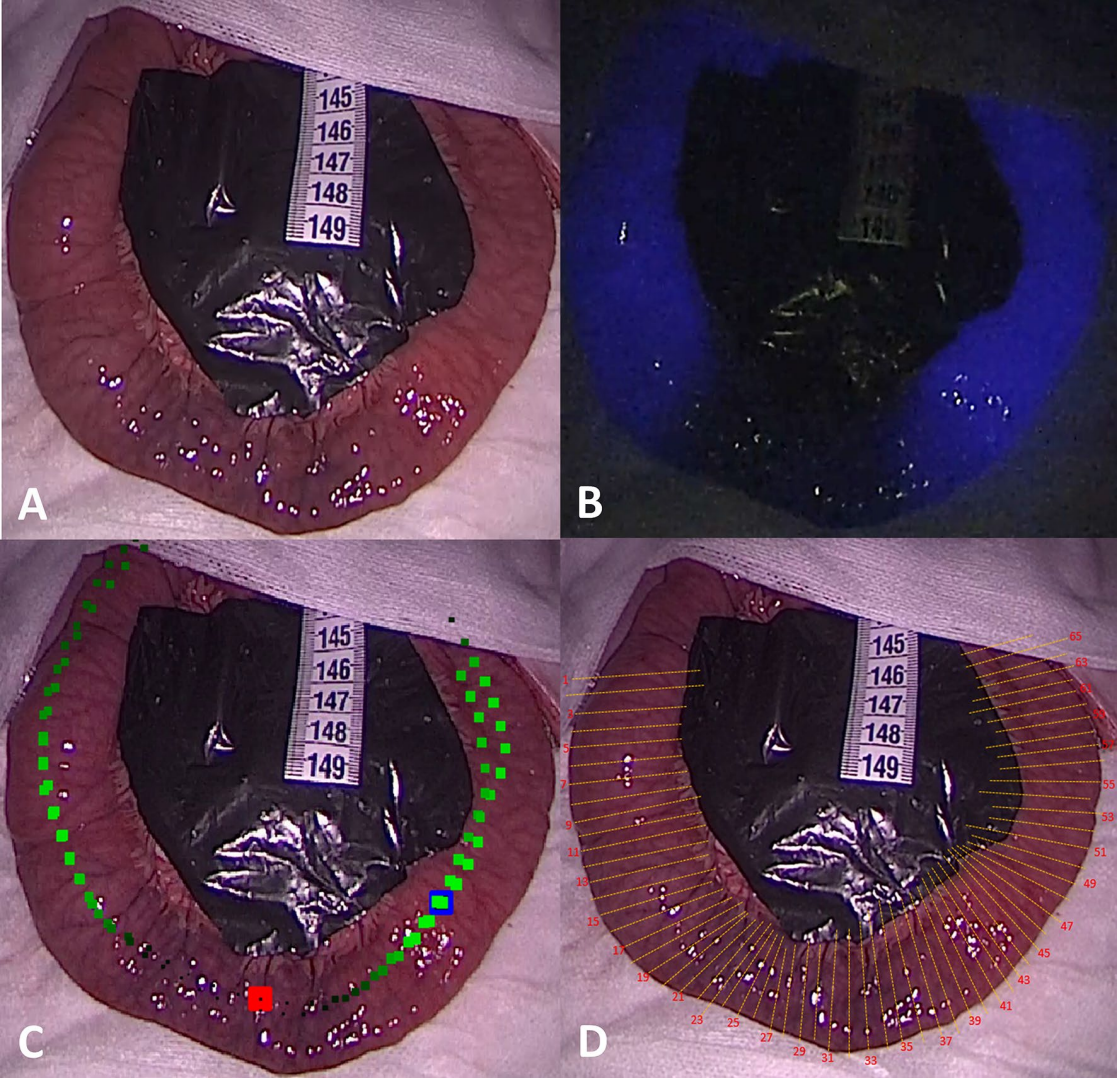
Examples of the laparoscopic images of partly devascularized small bowel with blinding of the mesentery presented to participants. **A** White light, **B** Indocyanine green angiography (both presented as video sequences). **C** Quantitative analysis of indocyanine green angiography, red indicates the lowest perfusion, black: low perfusion, blue: the maximal perfusion, and green: sufficient perfusion. **D** Corresponding image for selection of resection lines (Color figure online)

A laparotomy was performed. To estimate the accuracy of perfusion assessment, 11 small bowel loops were created in a total of five pigs, varying the degree of devascularization by ligating the feeding vessels. Also, two “sham” segments with no intervention, meaning normal perfusion, were included. The mesentery of all segments was hidden with black plastic to blind the observer to the degree of devascularization (Fig. 1).

### Perfusion assessment

Videos of the 13 bowel segments were recorded in WL and with ICG-FA using a laparoscopic camera and light source (Xenon) system (ICG-Hopkins 30°, D-Light P, Image 1 SPIES, Karl Storz GmbH and co., Tüttlingen, Germany). For ICG-FA a bolus of 0.25 mg/kg ICG (Verdye®, Diagnostic Green GmbH, Aschheim-Dornach, Germany) was injected in a peripheral vein and flushed with 5 ml of saline. All ambient light was turned off during the ICG-FA. Ventilation was paused for 10–20 s after injection of ICG to avoid respiratory movement artifacts during the quantification. For quantification, we used previously validated software (q-ICG) [13–17], and a picture of a color-coded output (beta-version of the present system [20]) representing relative perfusion of the segments (Fig. 1). This resulted in a total of 39 video and image combinations.

### Participants

With departmental and institutional ethical approval, participants with varying degrees of surgical experience from surgical departments in Denmark were recruited and asked to answer an online questionnaire to obtain demographic data. The participants were divided into three groups according to the level of experience, specifically by the number of intestinal resections they had performed unsupervised by a senior surgeon. All participants had to have at least assisted one intestinal resection. The groups were defined as novices: no unsupervised intestinal resections performed, intermediates: 1–49 unsupervised intestinal resections, and experienced: > 50 unsupervised intestinal resections.

### Data acquisition

Through the online questionnaire, the participants would download a slideshow, with the above-mentioned video and image combinations from WL, ICG-FA, and q-ICG. Along with the video/image, a WL image of the same bowel segment was provided with numbered resection lines (Fig. 1). The participants were then asked to choose two resection lines if they found ischemia or to note “no resection”. The numbered resection lines or “no resection” were written in the online questionnaire. The distance between these lines was measured digitally (ImageJ, v.1.47, U.S. National Institutes of Health, Bethesda, Maryland, USA) using the paper ruler for the conversion of the pixels measurements to metric units (mm).

To compare resection lines between groups and modalities, videos of ICG-FA from the true ischemic (non-sham) segments were re-analyzed with q-ICG. The sham segments were discarded in this analysis, as the correct answer was not to resect. Resection lines were set by the system at a conservative perfusion threshold of 70% of maximum perfusion in the field of vision. The threshold was based on a previous study on basis of the anastomotic tensile strength being significantly higher in anastomoses constructed with 100% and 60% relative perfusion compared with 30%, indicative of improved anastomotic healing [21]. The comparison of resection lines was done by comparing the participant’s selection with the automatic generated line, yielding both information about if participants had left ischemic tissue in situ (unsafe resection) or not (safe resection). A maximum of two lines away [median 8.8 mm (IQR 1.7 mm)] from the automatic generated line in the ischemic area was tolerated and considered a safe resection.

### Statistics

Histograms and Q-Q-plots were used to examine the normality of the distribution of data. Normally distributed data were reported as mean ± standard deviation (SD) and non-normally distributed data as median with range or interquartile range (IQR). Contingency tables were constructed to calculate the specificity, sensitivity, and positive/negative predictive value of the ability to detect ischemia under the different modalities. Contingency tables were also constructed to calculate chi-square (*X*^2^) and relative risk (RR) of leaving ischemic tissue in situ. *p*-values less than 0.05 were considered significant. Statistical analyses were done with IBM SPSS Statistics (v. 25, SPSS Inc, IBM, Chicago, IL, USA), and graphs were created in GraphPad Prism (GraphPad Software, San Diego, CA, USA).

## Results

We recruited a total of 45 participants, 18 novices, 12 intermediates, and 15 experienced (Table 1) and every participant completed the perfusion assessment (Table 1).

**Table 1:** Participant demographics

	Novices (*n* = 18)	Intermediates (*n* = 12)	Experienced (*n* = 15)
Sex, male/female	9/9	7/5	13/2
Age, years mean (± SD)	28.5 (± 3.9)	38.4 (± 4.9)	46.9 (± 10.5)
Years in surgery, years median (IQR)	0.5 (0–1.25)	7 (5–9.75)	16 (9–38.6)
Intestinal resections performed without supervision, median (IQR)	0	7.5 (2–40)	200 (150–1000)
ICG-knowledge, *n* (%)	3 (16.7%)	3 (25%)	8 (53.3%)

### Detection of sham segments with no ischemia

Using WL, the experienced surgeons (specificity: 100%, CI95% [89;100%]) were significantly better than novices (specificity 78%, CI95% [62;88%], *p* = 0.006) and intermediates (83%, CI95% [64;93%] p = 0.034) to correctly detect the sham segments with no ischemia. With the use of ICG-FA and q-ICG, the differences in specificity between groups diminished and became non-significant. The sensitivity and the negative predictive value improved in all groups when using q-ICG as compared with WL and ICG-FA alone. However, no differences in the positive predictive value between modalities and groups were found. The experienced group and the novices and intermediates seemed to improve in sensitivity to a minor degree but with some loss of specificity when using q-ICG (Table 2).

**Table 2:** Sensitivity, specificity and positive/negative predictive value of the ability to detect ischemia under the different modalities

	Sensitivity	Specificity	PPV	NPV
WL all	82.0 (78.4;85.2)	86.7 (78.1;92.2)	97.1 (95.1;98.4)	46.7 (39.3;54.3)
ICG all	85.3 (81.9;88.1)	97.8 (92.2;99.6)	99.5 (98.3;99.9)	54.7 (47.0;62.2)
q-ICG all	97.0 (95.1;98.2)	83.3 (74.3;89.6)	97.0 (95.1;98.2)	83.3 (74.3;89.6)
Novices				
WL	84.9 (79.2;89.2)	77.8 (61.9;88.3)	95.5 (91.3;97.7)	48.3 (35.9;60.1)
ICG	84.9 (79.2;89.2)	94.4 (81.9;0.99)	98.8 (95.8;99.8)	53.1 (41.1;64.8)
q-ICG	97.0 (93.6;98.6)	88.9 (94.9;99.2)	98.0 (94.9;99.2)	84.2 (69.6;92.6)
Intermediate				
WL	75.8 (67.8;82.3)	83.3 (64.2;93.3)	96.2 (90.5;98.5)	38.5 (26.5;52.0)
ICG	86.4 (79.5;91.2)	100 (86.2;100)	100 (96.7;100)	57.1 (42.2;70.1)
q-ICG	97.7 (93.5;99.4)	75.0 (55.1;88.0)	95.6 (90.6;98.0)	85.7 (65.4;95.0)
Experts				
WL	83.6 (77.2;88.5)	100 (88.7;100)	100 (97.3;100)	52.6 (39.9;65.0)
ICG	84.9 (78.6;89.5)	100 (88.7;100)	100 (97.3;100)	54.6 (41.5;67.0)
q-ICG	96.4 (92.3;98.32)	83.3 (66.4;92.7)	97.0 (93.1;98.7)	80.1 (63.7;90.8)

Values are percentages (CI95%)

*WL* White light, *ICG* indocyanine green, *q-ICG* quantitative indocyanine green, *PPV* Positive predictive value, *NNV* negative predictive value

### Safety

**Fig. 2:**
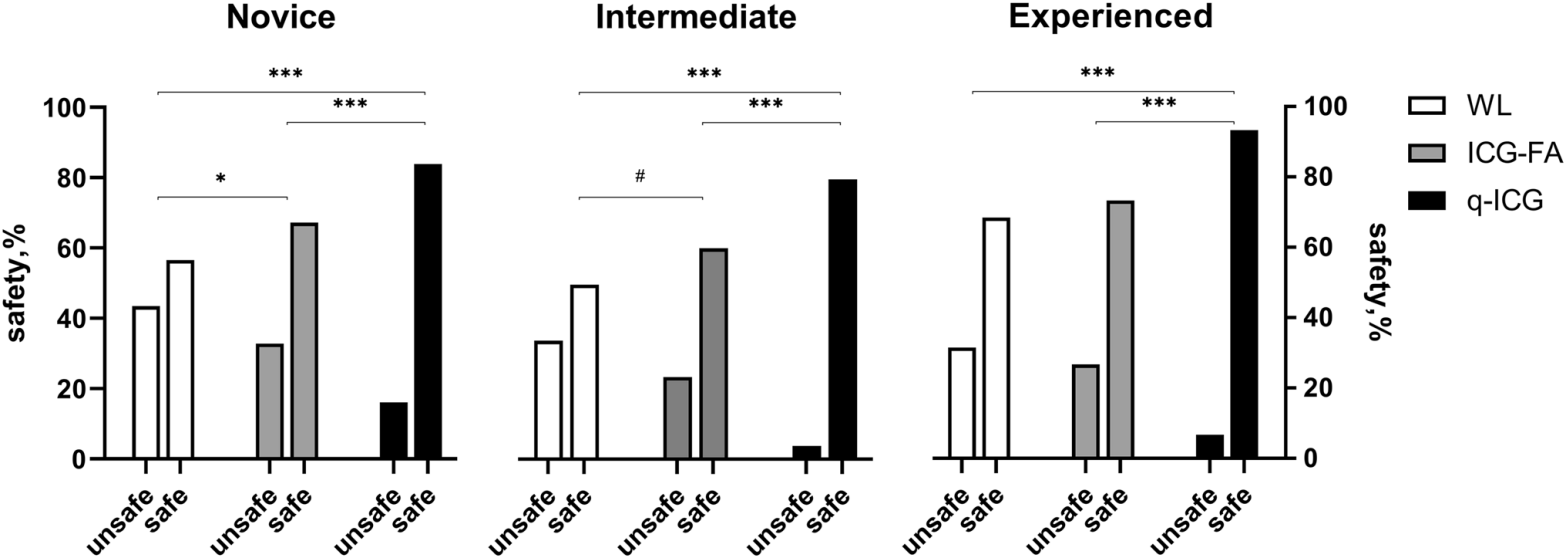
Chi-square of contingency tables of safe/unsafe resections across modalities in each experience group *p = 0.03, **p = 0.034, ***p < 0.001, #*p* = 0.034

**Fig. 3:**
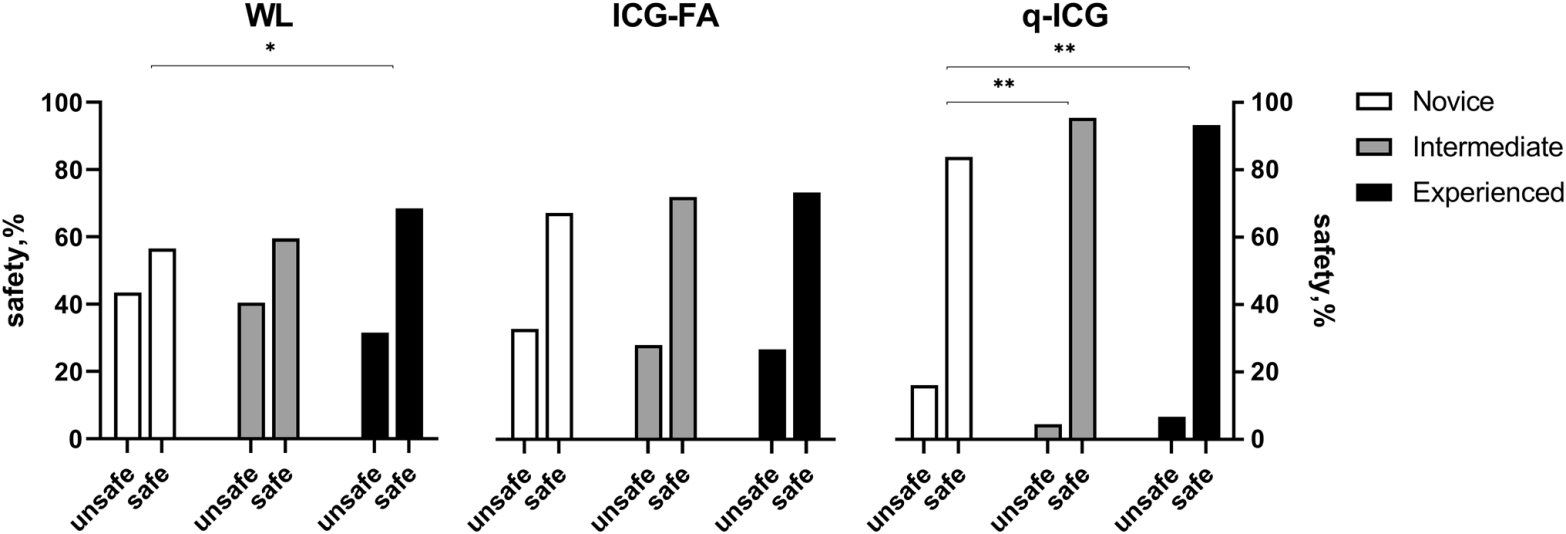
Chi-square of contingency tables of safe/unsafe resections across experience in each modality. *p = 0.020, **p = 0.005, ***p = 0.001

Regarding the safety, the experienced performed significantly safer resection than the novices (*p* = 0.02) when using WL. No differences between groups were proven when using ICG-FA, but with q-ICG, the experienced (*p* = 0.005) and intermediates (*p* = 0.001) performed significantly safer resections than novices. Novices and intermediates performed better by leaving significantly less ischemic tissue when using ICG (novices: *p* = 0.030; intermediates: *p* = 0.034) and q-ICG (novices*:* *p* < 0.001; intermediates: *p* = 0.001) compared with WL. The experienced did not benefit from ICG-FA, but with q-ICG, they improved in safety compared with both ICG (*p* < 0.001) and WL (*p* < 0.001) see Figs. 2 and 3.

The relative risk (RR) of performing an unsafe resection in WL was found significant for novices RR = 1.3, CI95% [1.0;1.7] and intermediates RR = 1.4, CI95% [1.0;2.0], but not for the experienced RR = 1.2, CI95% [0.8;1.7]. The RR for performing unsafe resection in WL was remarkably high when comparing with q-ICG in all groups; novices RR = 2.7, CI95% [1.9;3.8], intermediates RR = 8.9, CI95% [4.0;20.0], and experienced RR = 4.7, CI95% [2.6;8.7]. Also, when comparing WL with ICG-FA, the RR for performing an unsafe resection was found significant for novices RR = 1.3, [CI95% 1.0;1.7] and intermediates RR = 1.4, CI95% [1.0;2.0], but not for the experienced RR = 1.2, [CI95% 0.8;1.7]. Finally, all groups seemed more susceptible to perform unsafe resections when using ICG-FA compared with q-ICG; novices RR = 2.0, CI95% [1.4;2.9], intermediates RR = 6.2, CI95% [2.7;14.1] and experienced RR = 4.0, [CI95% 2.1;7.5].

## Discussion

In the present study, we found that ICG-FA and q-ICG helped novice surgeons improve sensitivity and specificity up to experienced surgeons' level when detecting non-ischemic bowel segments correctly in a blinded setup. Further, we found that ICG-FA and q-ICG significantly helped novices and intermediates perform safer resections and that even experienced surgeons significantly improved in safety when using q-ICG but not ICG-FA alone. A four- to nine-fold risk of leaving ischemic tissue in WL compared with q-ICG was found for intermediates and experienced. In WL the experienced performed safer resections than novices, but the difference diminished when novices were using ICG-FA. When q-ICG was used, almost all the intermediates and experienced performed exclusively safe resections, leading to a significant difference compared with the novice resections.

We found that novices performed 57% safe resections in WL compared with 67% with ICG-FA (*p* = 0.03) and 84% (*p* < 0.001) with q-ICG. Even more interesting, this tendency to perform safer resections when using ICG-FA and q-ICG was evident in all groups regardless of experience level (Fig. 2). Sensitivity and specificity have previously been investigated concerning perfusion assessment with and without ICG. In a study of 191 patients undergoing colorectal resection and anastomosis, the surgeons were asked to determine the risk of AL. The actual AL rate was 13.6%, and the median predicted rate was 7.1%. The sensitivity/specificity was calculated to 38/46% for high, and 62/52% for low anastomoses and no significant difference between assisting and consulting surgeons was found (*p* = 0.2) [7]. An experimental rodent study found that clinical perfusion assessment (using WL) had a high sensitivity for predicting the outcome of ischemic intestines of 93–100% but a relatively low specificity (47–70%), which increased with the use of ICG-FA (77–92%). The authors also implied quantitative measures, but these were inferior to the clinical and the visual assessment of ICG-FA [22].

The educational benefit of ICG-FA has been investigated in a study of 21 patients undergoing colorectal resection. Medical students (*n* = 11) and surgical residents (*n* = 11) were asked to determine resection lines on still pictures (WL, ICG-FA) of the resection site. The experienced surgeons (sample size not reported) modified the colonic transection line in three patients (14.3%), compared with 59.1% of patients judged by beginners (*p*-value not reported). The beginners had a mean accuracy of 39.4% in WL compared with 81.8% in ICG-FA (*p* < 0.05) [23]. This was confirmed by our results with safe resection of 57% in WL, 67% with ICG-FA, and 84% using q-ICG.

A recent study investigating the inter-user variation in interpretation of ICG-FA during surgical resection found that this variation is influenced not only by surgical experience but also by experience with ICG-FA. The study speculates that experienced surgeons may use ICG-FA only as an adjunct to confirm their surgical conclusions, and highly recommend further development of quantification methods [24]. Interestingly, we found that the relative risk of performing unsafe resection using ICG-FA compared with q-ICG was 2.0, 6.2, and 4.0 for novice, intermediates, and experienced. This supports the fact that the interpretation of ICG-FA may be subjective and with variations.

Our q-ICG algorithm uses an inflow parameter (slope of the fluorescence time-curve) instead of the intensity parameters (maximum fluorescence) included in some of the commercially available fluorescence-guided surgery equipment. A study of 86 patients undergoing colorectal resection analyzed different q-ICG parameters postoperative and found significant differences in inflow parameters (*p* = 0.001) between patients with and without AL. No significant difference was found when using intensity parameters. The area under the curve of predicting AL using inflow parameters was calculated to 0.93 by a certain cut-off value. Using this cut-off value a multivariate analysis resulted in an odds ratio of leakage of 130.8, CI95% [6.5;2654.8] [25]. Other studies have not reproduced this very substantial result, but a systematic review found that intensity parameters are unstable and unreliable in clinical settings [26]. Therefore, we urge surgeons to not rely on intensity parameters when exploring the use of quantitative ICG angiography.

A similar quantification algorithm (FLER) using an inflow parameter (“slope of the time-to-peak”) has been thoroughly validated in animal experimental studies [27–30] and in a prospective clinical trial [31]. The FLER algorithm has also been compared to our q-ICG algorithm in the analysis of perfusion in a porcine ischemic setup. Small, presumably clinical non-relevant, differences were found in low and high perfused areas [32].

There are still challenges to overcome before q-ICG may be a standard tool in gastrointestinal surgery. The consensus of which parameter to use remains to be settled, and cut-off values predicting leakage need to be investigated in large scale studies. Also, most studies have only used q-ICG postoperatively [26]. Nevertheless, intraoperative use of q-ICG is feasible and with excellent usability, leading to a change in the surgical resection site [20].

The additional information provided by ICG-FA and q-ICG may act as a “virtual supervisor” and thereby assist novices in surgical training to improve the accuracy of intraoperative perfusion assessment. The most senior surgical experience is not always available, and thus decisions to resect viably or leave non-viable tissue in the patient may be taken on a false basis. As AL is related to low anastomotic perfusion and has vast consequences for the patients, tools that may improve and objectify perfusion assessment is highly warranted [12, 33].

**Fig. 4:**
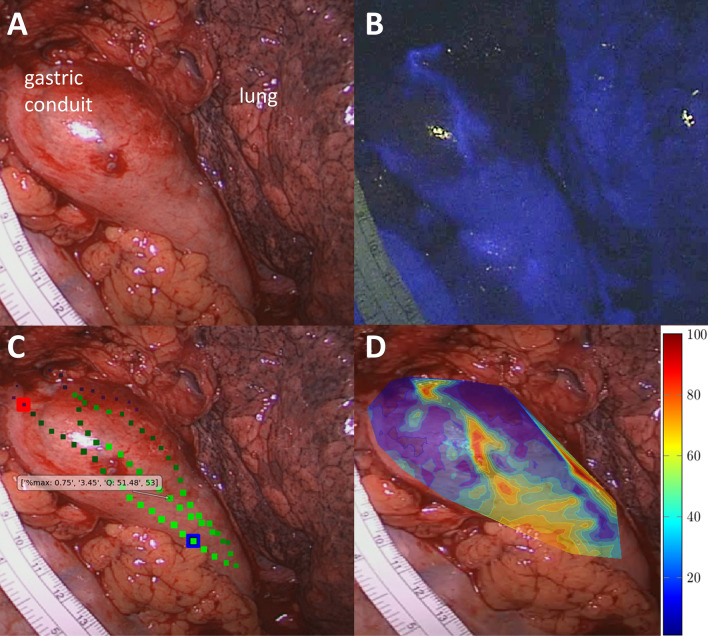
Example of tablet-based q-ICG used during the thoracic part of Ivor-Lewis resection of gastroesophageal cancer. **A** white light image, **B** Indocyanine green angiography, **C** Quantitative analysis of indocyanine green angiography with interactive features. **D** Color-coded heatmap derived from q-ICG representing relative perfusion. Adapted from [20] with permission from Springer and Langenbeck’s Archive of Surgery (Color figure online)

The present study has certain limitations. First, the study did not investigate clinical outcomes of eventual resection and anastomosis, but only if resection was done in perfused tissue or ischemic tissue as deemed by q-ICG posthoc. Nevertheless, another porcine experimental study has investigated the tensile strength of anastomoses constructed in 30, 60, and 100% perfusion using a similar q-ICG algorithm. The anastomoses made in 30% perfusion showed significantly lower tensile strength on postoperative day 5 than anastomoses of 60 and 100% perfusion [21]. Secondly, we used a preliminary beta-version of q-ICG to produce pictures of outputs to the participants (Fig. 1). More detailed and informative outputs are available today. The newest plug-in tablet-based system can provide the surgeon with exact interactive information of relative and absolute perfusion in several regions of interest and provide a color-coded heat map (Fig. 4) [20]. A combination of ICG-FA and q-ICG will presumably result in close to optimal sensitivity and specificity in real life.

In conclusion, we found that quantitative indocyanine green angiography seems to guide surgeons, regardless of their level of experience, to perform safer resections than when using standard WL or visual assessment of indocyanine green angiography. Further studies should focus on the clinical impact of the novel tool.
